# Glucosylation by the *Legionella* Effector SetA Promotes the Nuclear Localization of the Transcription Factor TFEB

**DOI:** 10.1016/j.isci.2020.101300

**Published:** 2020-06-20

**Authors:** Wendy H.J. Beck, Dongsung Kim, Jishnu Das, Haiyuan Yu, Marcus B. Smolka, Yuxin Mao

**Affiliations:** 1 Weill Institute for Cell and Molecular Biology, Cornell University, Ithaca, NY 14853, USA; 2 Department of Molecular Biology and Genetics, Cornell University, Ithaca, NY 14853, USA; 3 Department of Computational Biology, Cornell University, Ithaca, NY 14853, USA

**Keywords:** Molecular Biology, Microbiology

## Abstract

*Legionella pneumophila* is an intracellular pathogen that requires nutrients from the host for its replication. It has been shown that replicating *L. pneumophila* prefers amino acids as main sources of carbon and energy. The homeostasis of amino acids in eukaryotic cells is regulated by the transcription factor EB (TFEB), which translocates into the nucleus and activates genes for autophagy and lysosomal biogenesis. Here we show that the *Legionella* effector SetA causes a robust nuclear translocation of TFEB when exogenously expressed in mammalian cells and that the translocation is dependent on the glucosyltransferase activity of SetA. We further show that SetA directly glucosylates TFEB at multiple sites. Our findings of TFEB glucosylation by SetA may suggest an alternative strategy for exploiting host nutrients in addition to the control of host mTORC1 signaling by *L. pneumophila*. Our results provide further insight into the molecular mechanism of the delicate TFEB nuclear shuttling.

## Highlights

• The *Legionella* effector SetA causes robust nuclear enrichment of TFEB
• SetA directly glucosylates TFEB at multiple Ser/Thr sites
• Glucosylation of the S138 site by SetA prevents TFEB nuclear export
• Glucosylation near the S211 site of TFEB disrupts the binding between TFEB and 14-3-3

## Graphical Abstract

**Figure:**
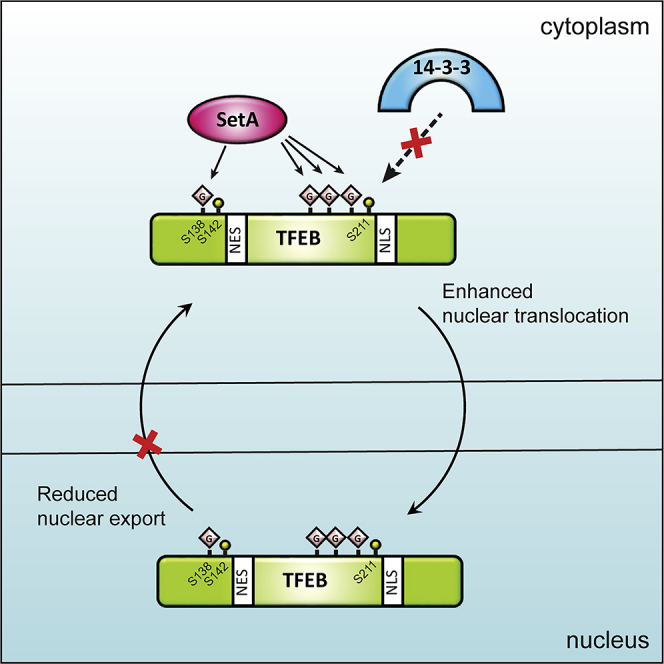

## Introduction

The gram-negative bacterium, *Legionella pneumophila*, is a facultative intracellular pathogen that parasitizes freshwater amoeba in nature (Fields, 1996). *L. pneumophila* can also replicate inside human alveolar macrophages upon the inhalation of bacteria-containing aerosols, causing a severe form of pneumonia known as Legionnaires' disease (Fraser et al., 1977; Horwitz and Silverstein, 1980). Upon entry into host cells, *L. pneumophila* releases a repertoire of ∼300 effector proteins through the Dot/Icm type IV secretion system to establish a replication niche, known as the *Legionella*-containing vacuole (LCV) (Hubber and Roy, 2010; Qiu and Luo, 2017). Like other intracellular bacterial pathogens, *L. pneumophila* relies on energy and anabolic substrates derived from the nutrient-limited intracellular environment for propagation. The level of nutrient availability is tightly coupled to the life cycle and virulence of *L. pneumophila* (Byrne and Swanson, 1998; Hammer et al., 2002; Hauslein et al., 2017; Oliva et al., 2018). In fact, accumulative evidence has documented that *L. pneumophila* utilizes a variety of strategies to stimulate nutrient supply from the host for optimal growth and replication.

A recurring strategy evolved by intracellular pathogens to increase nutrient availability is to leverage host degradative processes, such as autophagy and ubiquitin-proteasome system (Fonseca and Swanson, 2014; Steele et al., 2015). In *L. pneumophila*, among the large cohort of effectors, the F-box-containing AnkB catalyzes the assembly of K48-linked polyubiquitinated substrates on the LCV to fuel the host proteasome for the production of amino acids (Price et al., 2011). *L. pneumophila* also leverages the suppression of protein synthesis pathways as a form of nutritional virulence. As many as five *Legionella* effectors have been shown to inhibit host translation, thereby freeing amino acids for use by the intracellular bacteria (Fontana et al., 2011). Among these effectors, three glucosyltransferases (Lgt1–3) specifically glucosylate mammalian elongation factor eEF1A to block host protein synthesis (Belyi et al., 2006, 2008). Furthermore, the Lgt family of glucosyltransferases, as well as the SidE family of phosphoribosyl ubiquitin ligases, have recently been shown to manipulate the master metabolic regulator mTORC1 to promote the generation of free amino acids for bacterial consumption (De Leon et al., 2017). These findings indicate that *L. pneumophila* has evolved intricate and diverse strategies to cope with the nutrient demands requisite for intracellular replication.

In eukaryotes, intracellular amino acid levels are sensed and tightly controlled through signaling pathways centered around the mTORC1 complex (the mechanistic target of rapamycin complex 1) (Bar-Peled and Sabatini, 2014; Goberdhan et al., 2016). In the presence of nutrients, mTORC1 is active and phosphorylates one of its downstream substrates transcription factor EB (TFEB) to promote cytoplasmic sequestration via interactions with the regulatory protein 14-3-3 (Roczniak-Ferguson et al., 2012; Settembre et al., 2012). However, in response to amino acid limitation, the inhibition of mTORC1 and the concomitant activation of the phosphatase calcineurin trigger dephosphorylation and nuclear translocation of TFEB (Medina et al., 2015). Upon entering the nucleus, TFEB rapidly activates the expression of lysosomal and autophagosomal genes, which boosts the number and activity of degradative organelles to break down host macromolecules for nutrient supply (Sardiello et al., 2009; Settembre et al., 2012).

In this study, we aimed to identify the potential strategies that *L. pneumophila* may utilize to acquire nutrients from the host to facilitate its intracellular proliferation. We identified several *Legionella* effectors that are able to override the phosphorylation signals on TFEB imposed by mTORC1, resulting in the nuclear translocation of TFEB. Particularly, we found that the *Legionella* effector, SetA, which was predicted as a glucosyltransferase based on shared sequence homology with other *Legionella* glucosyltransferases, promotes TFEB nuclear localization in a glucosyltransferase activity-dependent manner. Mass spectrometry (MS) analysis further revealed that SetA modifies TFEB at several sites adjacent to the 14-3-3 phosphosite (S211) and a GSK3β phosphosite (S138). TFEB glucosylation by SetA not only disrupts the interaction of TFEB with 14-3-3 but also interferes with its export from the nucleus and thus causes nuclear retention of TFEB. Together, our results suggest a potential strategy that *L. pneumophila* may have evolved to acquire nutrients from the host and also shed light on the molecular mechanism of the nuclear import-export cycle of TFEB.

## Results

### A Screen for *Legionella* Effectors Perturbing the Intracellular Localization of TFEB

The intracellular localization of TFEB has been used as a readout for cellular nutritional states. We utilized a HeLa cell line stably expressing TFEB-GFP (Roczniak-Ferguson et al., 2012) as a reporter to screen for *Legionella* effectors that can perturb the intracellular localization of TFEB. We first generated a library containing 319 effectors from *L. pneumophila* strain Philadelphia 1 fused to an N-terminal mCherry tag. DNA constructs from the library were than transfected into the TFEB-GFP HeLa cells individually, and the intracellular localization of TFEB-GFP was analyzed by fluorescence microscopy. Through this imaging-based screen, we identified several effectors that induced constitutive TFEB nuclear localization under normal growth conditions (Figure S1). As a validation of our findings, two effectors that induced the nuclear translocation of TFEB, *sdeA* and *sdeC*, which encode enzymes that catalyze a unique type of phosphoribosyl ubiquitination independent of E1 or E2 enzymes (Bhogaraju et al., 2016; Kotewicz et al., 2017; Qiu et al., 2017), were also reported to induce nuclear translocation of TFEB in a similar screen (De Leon et al., 2017). However, other positive hits identified in our screen have not been reported in previous studies. These effectors include *setA*, a glucosyltransferase that modulates host cell membrane trafficking pathways (Belyi et al., 2011; Heidtman et al., 2009; Wang et al., 2018); *vipD*, a Rab5-activated phospholipase A1 that inhibits endosomal fusion (Gaspar and Machner, 2014; Lucas et al., 2014; Shohdy et al., 2005); and several other effectors with unknown function (*lpg2425*, *lpg2828*, and *lpg2888*). We focused on the effector SetA due to its well-defined enzymatic activity and its high efficiency to trigger TFEB nuclear translocation with minimal cell death when exogenously overexpressed in our screen.

### The Glucosyltransferase Activity of SetA Is Responsible for the Nuclear Translocation of TFEB

**Figure 1:**
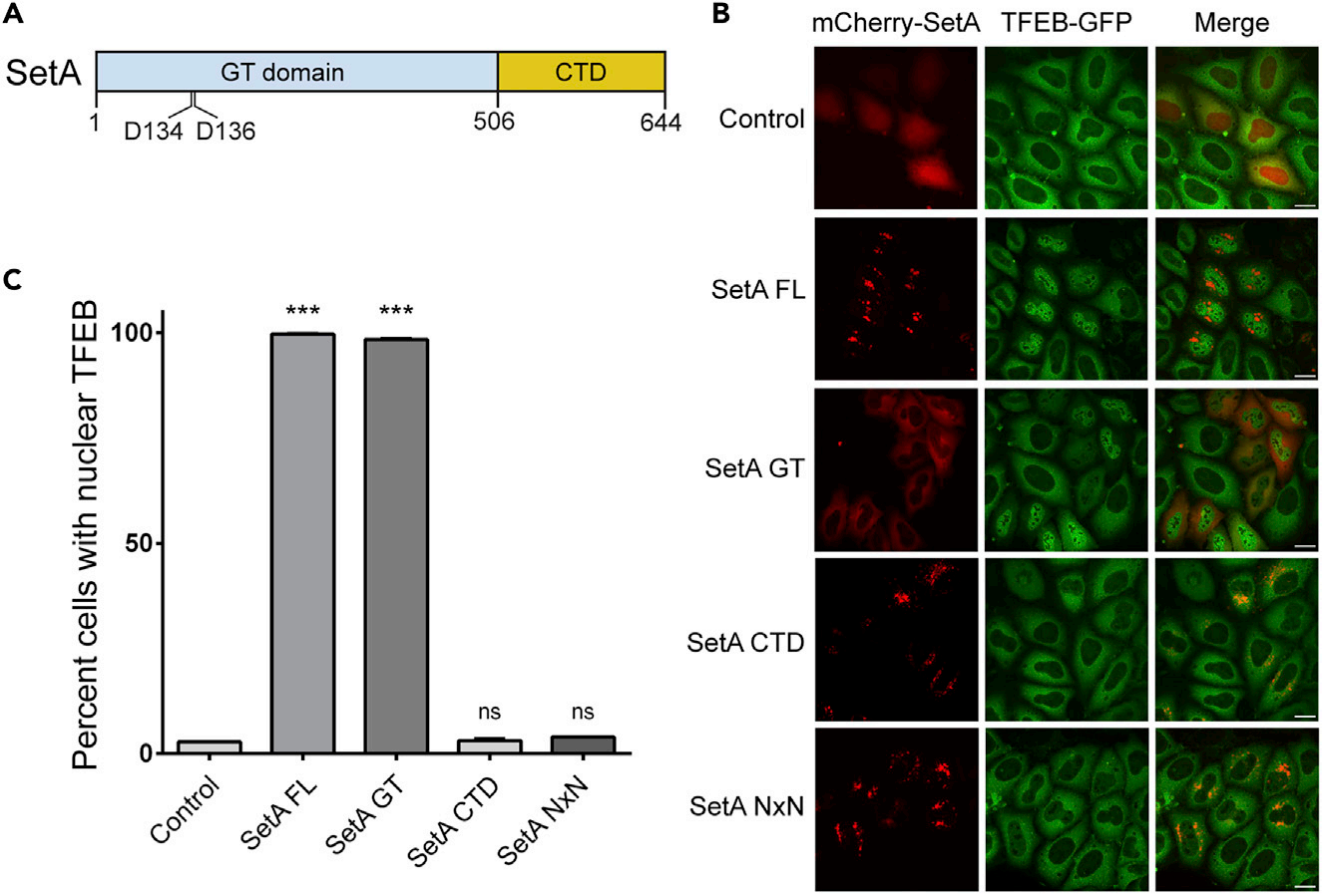
SetA Induces Nuclear Translocation of TFEB via Its Glucosyltransferase Domain (A) Schematic diagram of SetA. SetA contains a glycosyltransferase domain (GT; light blue) and C-terminal PI(3)P binding domain (CTD; orange). Two catalytic residues, D134 and D136 are labeled. (B) Representative images of TFEB-GFP stable cells transfected with plasmids expressing indicated SetA constructs. Scale bars, 20 μm. (C) Quantification of percentage of cells with nuclear TFEB-GFP in cells transfected with SetA constructs as in (B). Quantification analysis representative of 3 replicate datasets; minimum of 100 cells per construct per dataset. ∗∗∗p < 0.001. Data are represented as mean ± SEM. See also Figure S1.

SetA was previously characterized to have two functional domains: an N-terminal glucosyltransferase domain, which attaches a glucose moiety to its targets from UDP-glucose, and a C-terminal PI(3)P (phosphatidylinositol 3-phosphate)-binding domain that is essential for the targeting of SetA to endosomes of host cells (Jank et al., 2012) (Figure 1A). To determine which domain of SetA is required to trigger the nuclear localization of TFEB, we generated mCherry fusions of the N-terminal glucosyltransferase domain, SetA-GT (1–506), and the C-terminal PI(3)P-binding domain, SetA-CTD (507–644). Although SetA-GT showed a diffuse cellular localization, it was able to trigger a robust TFEB nuclear translocation comparable to full-length SetA. However, SetA-CTD failed to induce TFEB nuclear translocation, even though it exhibited a similar punctate localization as full-length SetA (Figures 1B and C). We next asked whether the catalytic activity of SetA is responsible for TFEB nuclear translocation. We generated a catalytically dead SetA mutant, SetA-NxN, which contains point mutants at two key catalytic residues (D134N and D136N) (Figure 1A). When expressed in the TFEB-GFP-stable HeLa cells, this mutant was unable to induce nuclear translocation of TFEB, although it had a similar localization as wild-type SetA (Figures 1B and 1C). Together, our data demonstrated that the glucosyltransferase activity of SetA is sufficient to induce TFEB nuclear translocation.

### SetA Disrupts the Binding between TFEB and 14-3-3 Independent of mTORC1 Activity

**Figure 2:**
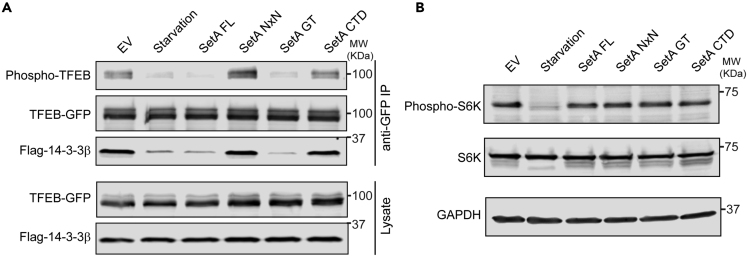
SetA Disrupts the Binding between TFEB and 14-3-3 Independent of mTORC1 Activity (A) Western blot analysis of anti-GFP coimmunoprcepitations (coIPs) from HEK293T cells transfected with plasmids expressing FLAG-14-3-3β, TFEB-GFP, and indicated mCherry-SetA constructs. The coIP FLAG-14-3-3β was blotted and detected by an anti-FLAG antibody, and phosphorylation status of TFEB S211 was detected using a phospho-14-3-3 motif antibody. (B) A stable TFEB-GFP HeLa cell line was transfected with the indicated mCherry-SetA expression constructs, and cell lysates were prepared and probed for total and phospho-S6K levels (T389) under the indicated experimental conditions. See also Figure S2.

Under nutrient-rich conditions, mTOR-dependent phosphorylation at serine 211 (S211) of TFEB promotes its association with 14-3-3 proteins, resulting in the retention of TFEB in the cytoplasm (Roczniak-Ferguson et al., 2012). We hypothesized that the activity of SetA may disrupt the interaction between TFEB and 14-3-3 to cause TFEB nuclear translocation. To test this hypothesis, we co-transfected TFEB-GFP and FLAG-14-3-3β in HEK293T cells along with an mCherry plasmid control or mCherry-SetA (Figure S2). FLAG-14-3-3β was able to coimmunoprecipitate with TFEB-GFP under normal growth conditions, whereas the interaction was largely reduced under amino acid starvation conditions (Figure 2A). Strikingly, both wild-type SetA and SetA-GT caused a significant disruption of this interaction in cells that were grown under nutrient-rich conditions, whereas no disruption was observed in the presence of SetA-NxN or SetA-CTD (Figure 2A). In addition, a decrease in S211 phosphorylation (phospho-TFEB) was observed in the presence of SetA constructs where loss of 14-3-3 binding was evident (Figure 2A).

As the interaction between TFEB and 14-3-3 is regulated by mTORC1 phosphorylation of TFEB, we next asked whether SetA interferes with mTORC1 activity. To assess mTORC1 kinase activity, we analyzed the phosphorylation status of an mTORC1 substrate, the ribosomal protein S6 kinase 1 (S6K1) (Burnett et al., 1998; Isotani et al., 1999). S6K1 phosphorylation was greatly reduced in amino acid-starved cells, due to the inactivation of mTORC1 (Figure 2B). However, the phosphorylation status of S6K1 was not affected by the expression of SetA constructs, indicating that SetA does not interfere with mTORC1 activity (Figure 2B). Together, our data suggest that the glucosyltransferase activity of SetA disrupts the interaction between TFEB and 14-3-3 proteins independent of mTORC1 activity.

### SetA Glucosylates TFEB at Multiple Sites

**Figure 3:**
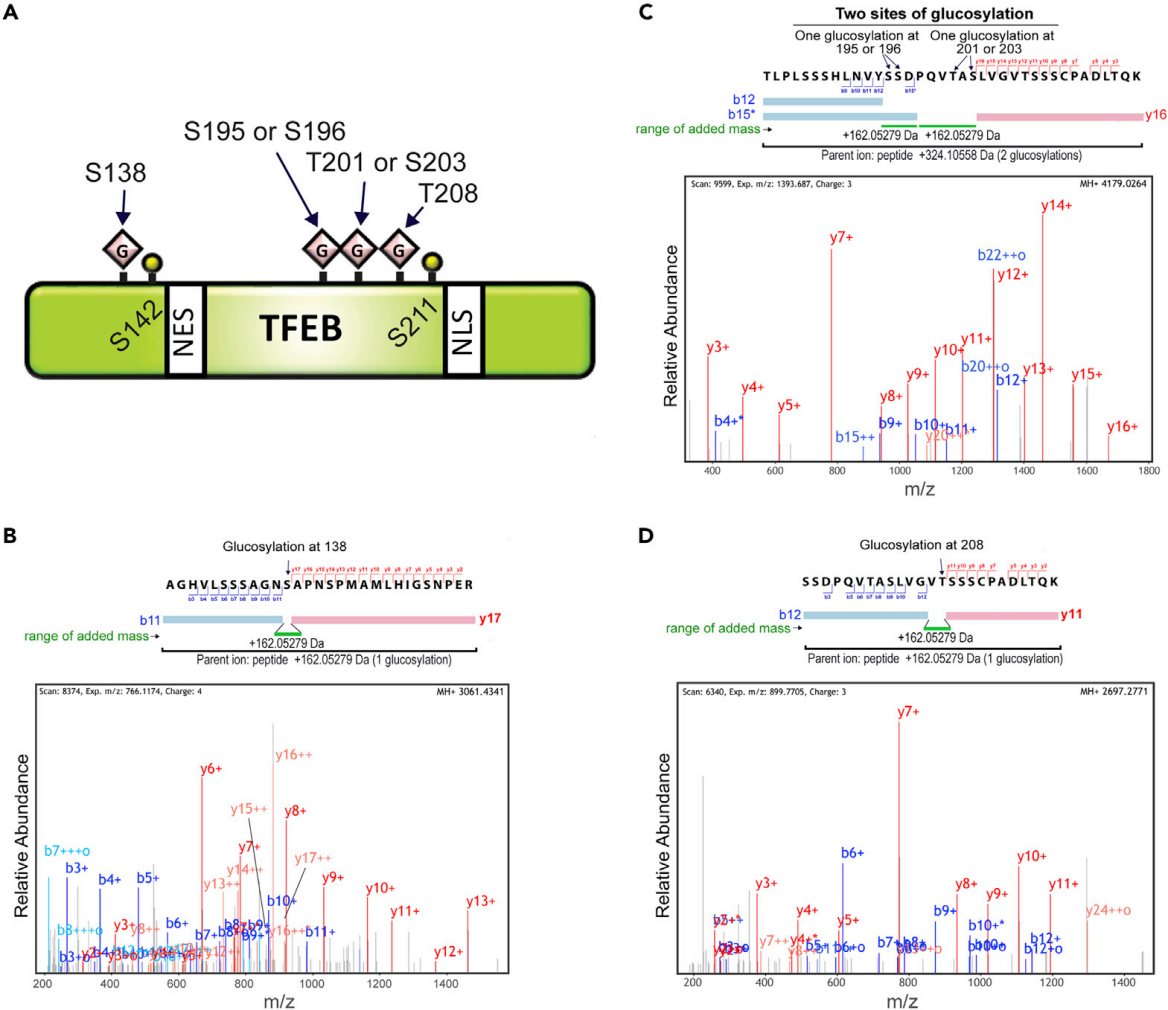
MS/MS Analysis of TFEB Glucosylation by SetA (A) Schematic diagram of TFEB with indicated SetA glucosylation sites that were identified by MS/MS. (B–D) MS/MS spectra for TFEB peptides carrying glucosylation sites identified in cells co-expressing TFEB and SetA. One glucosylation site was identified at S138 (B), two glucosylation sites were identified at S195 or S196 and T201 or S203 (C), and one glucosylation site was identified at T208 (D). The green bars indicate the peptide range with additional mass corresponding to one glucose.

As SetA disrupts the interaction between TFEB and 14-3-3 and triggers TFEB nuclear translocation without the intervention of mTORC1, we speculated that SetA may modify TFEB and/or 14-3-3 to cause disruptions in binding. To test this possibility, we used SILAC (stable isotope labeling by amino acids in cell culture) MS approach. HEK293T cells grown in medium containing “heavy” lysine (^13^C_6_, ^15^N_2_) and arginine (^13^C_6_, ^15^N_4_) were co-transfected with mCherry-SetA-GT, TFEB-GFP, and FLAG-14-3-3β, whereas cells grown in “light” (normal) lysine (^12^C_6_, ^14^N_2_) and arginine (^12^C_6_, ^14^N_4_) medium were co-transfected with mCherry-SetA-GT-NxN, TFEB-GFP, and FLAG-14-3-3β. TFEB-GFP was enriched by immunoprecipitation using GFP nanobody-conjugated resins, whereas FLAG-14-3-3β was enriched with anti-FLAG antibody-conjugated resins. Although no modifications were detected for 14-3-3, tandem MS (MS/MS) analysis of immunoprecipitated TFEB-GFP revealed multiple glucosylated sites on TFEB found exclusively in cells expressing wild-type SetA (Figure 3A). These modified sites include a GSK3β phosphosite, S138 (Figure 3B), and a Ser/Thr cluster, encompassing residues S195, S196, T201, S203, and T208, adjacent to the 14-3-3 binding site (Figures 3C and 3D).

### Glucosylation at S138 by SetA Impairs TFEB Nuclear Export

**Figure 4:**
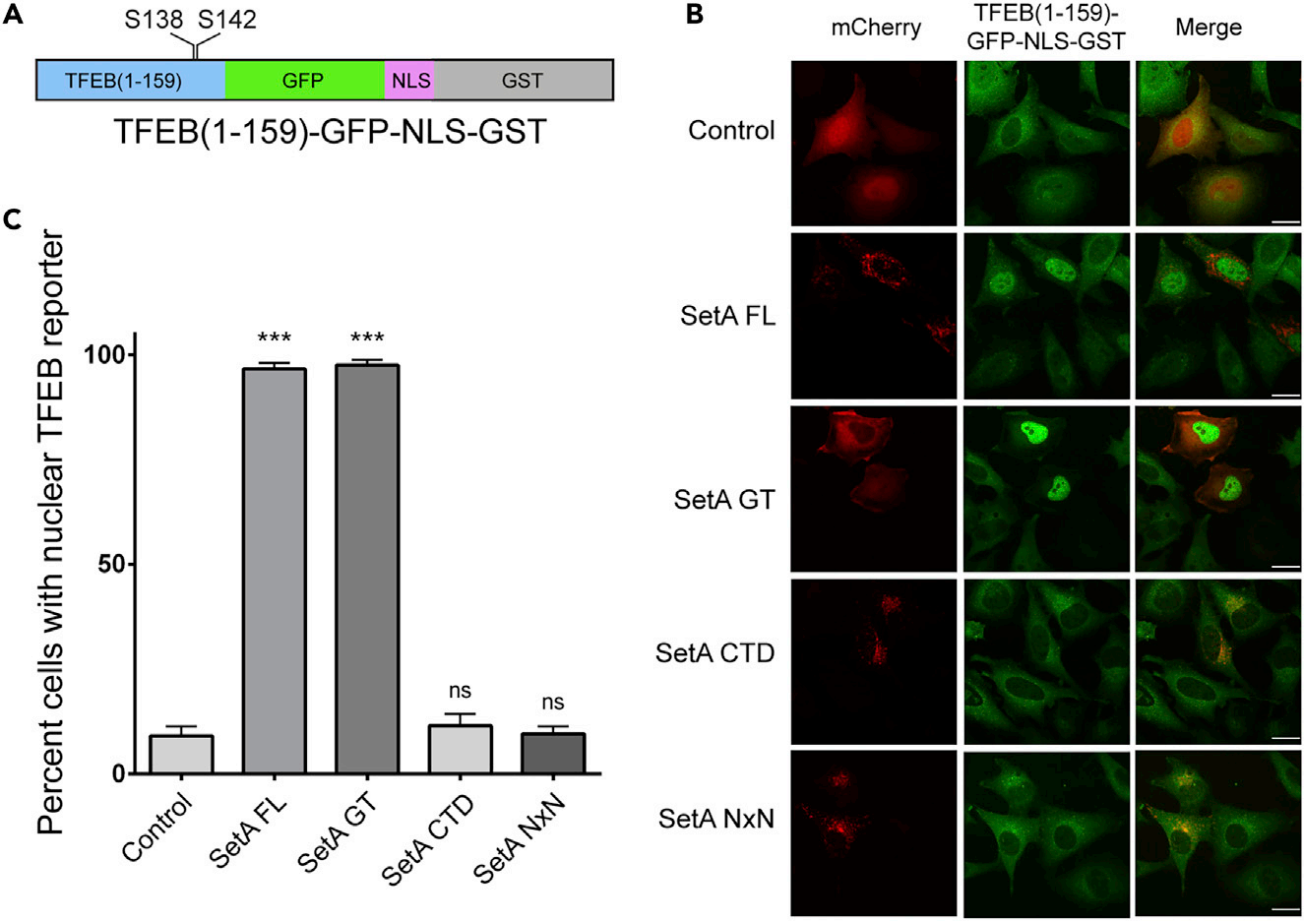
SetA Disrupts Activity of the TFEB Nuclear Export Signal (A) Schematic diagram of an artificial TFEB-NES GFP-NLS-GST reporter construct. (B) Representative images of a stable TFEB-NES-GFP-NLS-GST HeLa cell line expressing transfected mCherry vector or mCherry-SetA constructs. Scale bars, 20 μm. (C) Percentage of transfected cells with nuclear TFEB NES reporter under treatment conditions as in (B). Quantification analysis representative of 4 replicate datasets; minimum of 50 cells per construct per dataset. ∗∗∗ p < 0.001. Data are represented as mean ± SEM. See also Figure S3.

We next sought to elucidate which glucosylation site(s) on TFEB is(are) responsible for SetA-induced TFEB nuclear localization. The S138 site of TFEB was previously reported to be phosphorylated by GSK3β and was implicated in promoting cytoplasmic retention of TFEB (Li et al., 2016). Recent studies showed that phosphorylation of both S138 and S142, two residues located in proximity of a nuclear export signal (NES), were necessary for TFEB nuclear export in a CRM1-dependent manner (Li et al., 2018; Napolitano et al., 2018). We hypothesized that TFEB glucosylation at S138 by SetA may disrupt the signal required for nuclear export and thus cause enrichment of TFEB in the nucleus. To test this hypothesis, we generated a HeLa cell line stably expressing a TFEB nuclear export reporter, TFEB (1–159)-GFP-NLS-GST (Figure 4A) (Li et al., 2018). This reporter is primarily localized in the cytoplasm; however, it is enriched in the nucleus upon treatment with leptomycin B, a bacterial metabolite that disrupts interactions between the CRM1 exportin complex and substrate nuclear export sequences (Figure S3) (Kudo et al., 1999; Li et al., 2018). Interestingly, this TFEB reporter exhibited strong nuclear localization in cells transfected with wild-type SetA or SetA-GT. Conversely, no nuclear enrichment of the reporter was observed in cells expressing SetA-NxN mutant or SetA-CTD (Figures 4B and 4C). These results suggest that SetA-mediated glucosylation of TFEB at S138 disrupts the signal for TFEB nuclear export and that this modification contributes to the nuclear retention of TFEB.

### SetA-Mediated Glucosylation near TFEB 14-3-3 Binding Site Disrupts Its Binding with 14-3-3

**Figure 5:**
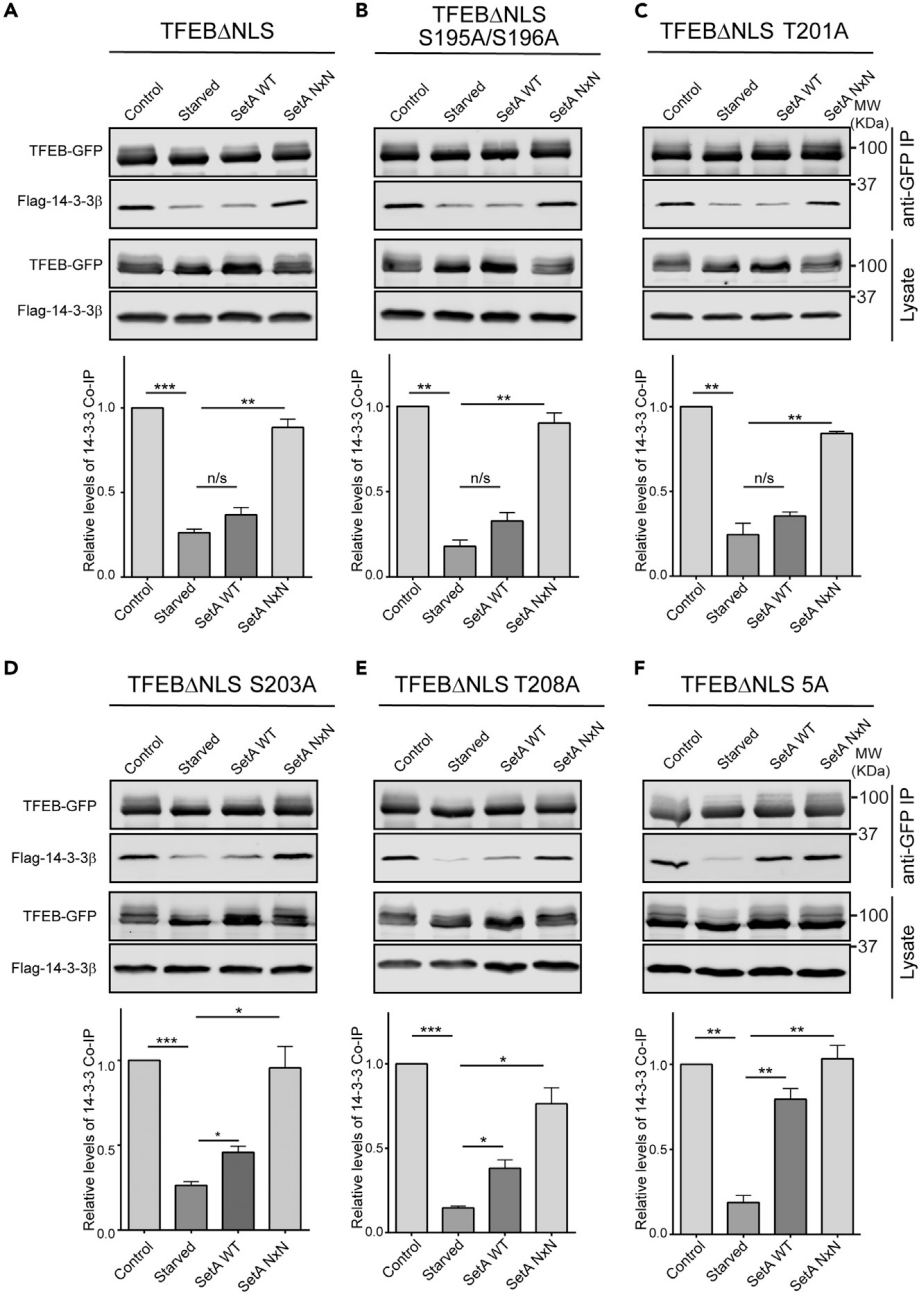
Assessment of SetA Modifications of Ser/Thr Residues Near 14-3-3 Binding Site (A) Western blot analysis of anti-GFP coimmunoprecipitations (coIPs) from HEK293T cells transfected with plasmids expressing indicated mCherry-SetA construct, FLAG-14-3-3β, and TFEBΔNLS-GFP. The coIP FLAG-14-3-3β was blotted and detected by an anti-FLAG antibody (top panel). Quantification of relative FLAG-14-3-3β levels coIP from 3 independent experiments. Data are shown as means ± SEM (bottom panel). (B–F) Similar western blot analysis of samples prepared from cells expressing indicated mCherry-SetA construct, FLAG-14-3-3β, and TFEBΔNLS-GFP carrying mutations at S195A/S196A (B), T201A (C), S203A (D), T208A (E), and alanine substitutions at S195, S196, T201, S203, and T208 (TFEBΔNLS 5A) (F). ∗p < 0.05, ∗∗p < 0.01, ∗∗∗p < 0.001. Data are represented as mean ± SEM. See also Figures S4 and S5.

In addition to S138, our MS/MS analysis detected a cluster of Ser/Thr residues near the 14-3-3 binding site on TFEB modified by SetA. We hypothesized that glucosylation near the TFEB 14-3-3 binding site impairs its interaction with 14-3-3. However, a TFEB mutant carrying alanine substitutions at S195, S196, T201, S203, and T208 (TFEB 5A) remained sensitive to SetA (Figure S4). We reasoned that the sensitivity may be due to the modification at S138. In fact, glucosylation at S138 by SetA was sufficient to disrupt the nuclear export signal of TFEB (Figure 4). Furthermore, an S138A or S142A mutant decreases the level of interaction between TFEB and 14-3-3 by causing a reduction in S211 phosphorylation (Figure S5A). To overcome the interference caused by S138 glucosylation, we introduced a TFEB mutant with its nuclear localization sequence (NLS) mutated (TFEBΔNLS-GFP) (Roczniak-Ferguson et al., 2012). This TFEB construct was resistant to perturbations at S138 and exhibited a level of S211 phosphorylation and 14-3-3 binding comparable to wild-type TFEB under normal growth conditions (Figure S5B). To identify which residue is responsible for the impaired binding with 14-3-3 upon glucosylation by SetA, we generated a series of alanine substitutions at positions S195, S196, T201, S203, and T208 in the context of the TFEBΔNLS-GFP construct. We found that single or double mutations of the five potential glucosylation sites were not sufficient to restore binding between TFEB and 14-3-3 in the presence of SetA (Figures 5A–5E). However, a TFEB mutant carrying alanine mutations at all five sites (TFEBΔNLS 5A) restored binding between TFEB and 14-3-3 in the presence of SetA and is thereby resistant to the glucosyltransferase activity of SetA (Figure 5F). These results suggest that SetA modifies multiple sites near the 14-3-3 binding motif on TFEB and that glucosylation by SetA in this region impedes the direct interaction between TFEB and 14-3-3.

## Discussion

The *Legionella* effector, SetA (lpg1978), was first established as a mono-*O-*glucosyltransferase that harbors a characteristic DxD catalytic motif and preferentially utilizes UDP-glucose as a sugar donor (Jank et al., 2012). However, the cellular targets of SetA were largely unknown until recently (Gao et al., 2019; Levanova et al., 2019; Wang et al., 2018). Several host components modified by SetA, including actin, vimentin, the chaperonin CCT5, and the small GTPase Rab1a, have diverse structures and functions. Our results, together with those of previous studies, suggest that SetA modifies a large array of cellular substrates and thus may modulate multiple cellular pathways, such as cellular cytoskeleton, membrane trafficking, and transcription regulation.

Our MS/MS analysis revealed that SetA modifies TFEB at two key regions, a GSK3β phosphosite (S138) and a cluster of serine and threonine (S/T) residues adjacent to the 14-3-3 binding site (S211). Using a TFEB (1-159)-GFP-NLS-GST reporter, we found that SetA-mediated glucosylation on S138 is sufficient to cause nuclear enrichment of the reporter. This finding corroborates recent discoveries that hierarchical phosphorylation on TFEB S138 and S142 activates an adjacent NES and promotes TFEB nuclear export (Li et al., 2018; Napolitano et al., 2018). Glucosylation on S138 by SetA likely precludes GSK3β-mediated phosphorylation on this site and thus causes TFEB nuclear retention by hampering TFEB nuclear export. We further examined the impact of SetA glucosylation on the S/T cluster near the S211 site. We found that TFEB mutants harboring single alanine substitution of S/T in this cluster were still unable to bind with 14-3-3 in the presence of SetA. However, a TFEB mutant carrying alanine substitutions at all five potential SetA targeting sites fully restored binding with 14-3-3 in the presence of SetA. These observations suggest that multiple S/T residues in this cluster are responsible for the loss of binding between TFEB and 14-3-3 upon modification by SetA. A recent structure of 14-3-3 in complex with a TFEB phospho-peptide encompassing S211 revealed that residues upstream of S211, including T208, make several contacts with the target-binding groove of 14-3-3 (Xu et al., 2019). It is likely that the addition of multiple bulky glucose moieties within the region upstream of S211 may sterically obstruct the interaction between the S211 site and 14-3-3. However, it may also be possible that SetA modifications of the S/T cluster near the S211 site interferes with S211 phosphorylation by mTORC1. Future experiments are required to pinpoint the exact role of SetA-mediated modifications at this S/T cluster. Nevertheless, the identification of two key regions on TFEB modified by SetA sheds light on the molecular mechanisms dictating TFEB nuclear shuttling.

To support growth in the host cellular environment, intracellular pathogens must acquire nutrients from the host itself. In *L. pneumophila*, several effectors play a role in facilitating bacterial acquisition of host amino acids by either boosting host degradative processes or inhibiting host protein synthesis. Emerging evidence indicates that host mTORC1 is a key target of *Legionella* for nutrient regulation, shared with other intracellular pathogens (Abshire et al., 2016; De Leon et al., 2017). In this study, we found that when overexpressed in mammalian cells, the *L. pneumophila* effector SetA directly glucosylates TFEB to cause nuclear retention. We further showed that SetA-mediated TFEB modification bypasses mTORC1 signaling for TFEB activation. However, future studies are required to address whether or not SetA directly targets endogenous TFEB under infection conditions. Nevertheless, our findings provide a potential strategy that might be applied by *L. pneumophila* to cope with its nutrient demands and further indicate the complexity and redundancy of such strategies encoded in intracellular pathogens.

### Limitations of the Study

In this study, we show that SetA glucosylates TFEB at multiple sites to cause nuclear localization and activation. Our work highlights a potential strategy that *L. pneumophila* utilizes for increasing amino acid production to support intracellular replication. However, the experiments tested herein use exogenously overexpressed SetA. Future work involving infection studies would be needed to determine if TFEB is a physiological target of SetA.

### Resource Availability

#### Lead Contact

Further information and requests for resources and reagents should be directed to and will be fulfilled by the Lead Contact, Dr. Yuxin Mao (ym253@cornell.edu).

#### Materials Availability

Reagents generated from this study are available upon request.

#### Data and Code Availability

The authors declare that all supporting data can be found in the article and the accompanying Supplemental Information.

## Methods

All methods can be found in the accompanying Transparent Methods supplemental file.

## Supplemental Information

Document S1. Transparent Methods, Figures S1–S5, and Table S1

## Footnotes

Supplemental Information can be found online at https://doi.org/10.1016/j.isci.2020.101300.
