# Supplementary material for: Glucosylation by the Legionella Effector SetA Promotes the Nuclear Localization of the Transcription Factor TFEB
Source: iScience. 2020 Jun 20;23(7):101300. doi: 10.1016/j.isci.2020.101300 (PMC7334434; doi:10.1016/j.isci.2020.101300)
Supplement: Document S1. Transparent Methods, Figures S1–S5, and Table S1 [file mmc1.pdf]

iScience, Volume *23*

Supplemental Information

Glucosylation by the *Legionella* Effector SetA Promotes the Nuclear Localization of the Transcription Factor TFEB

Wendy H.J. Beck, Dongsung Kim, Jishnu Das, Haiyuan Yu, Marcus B. Smolka, and Yuxin Mao

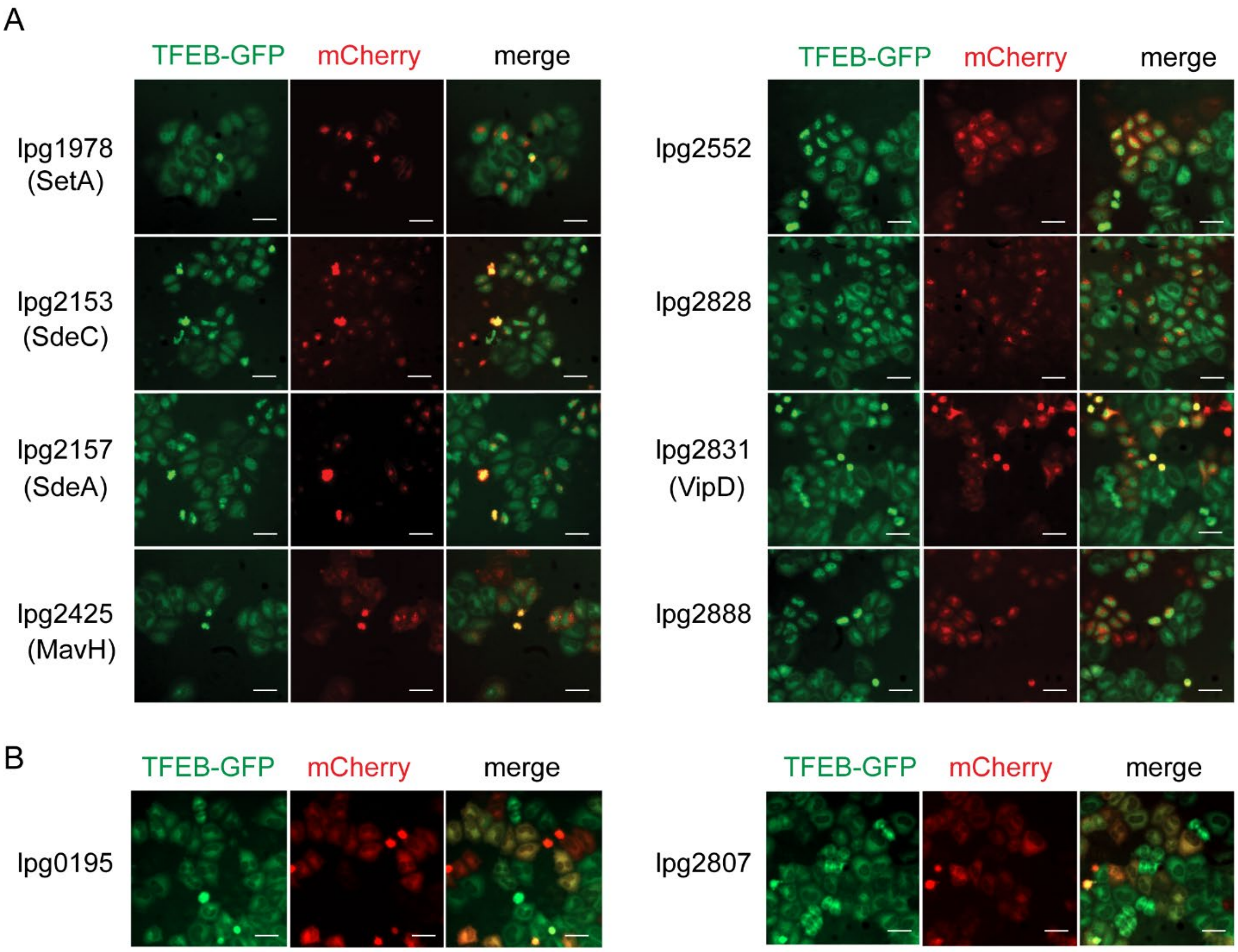

Figure S1, related to Figure 1. Screen hits of *L. pneumophila* effectors that cause nuclear translocation of TFEB. (A) Representative images of effectors identified to induce robust nuclear enrichment of TFEB, and (B) two selected effectors that do not disrupt TFEB localization from the screen using the effector protein library generated from *L. pneumophila* strain Philadelphia 1. A stable TFEB-GFP expressing HeLa line was transfected with individual *Legionella* effectors fused to a N-terminal mCherry tag in 96-well plates. Cells were fixed 14-16 hours post-transfection and imaged in-well using fluorescence microscopy. Scale bar represents 50 μm.

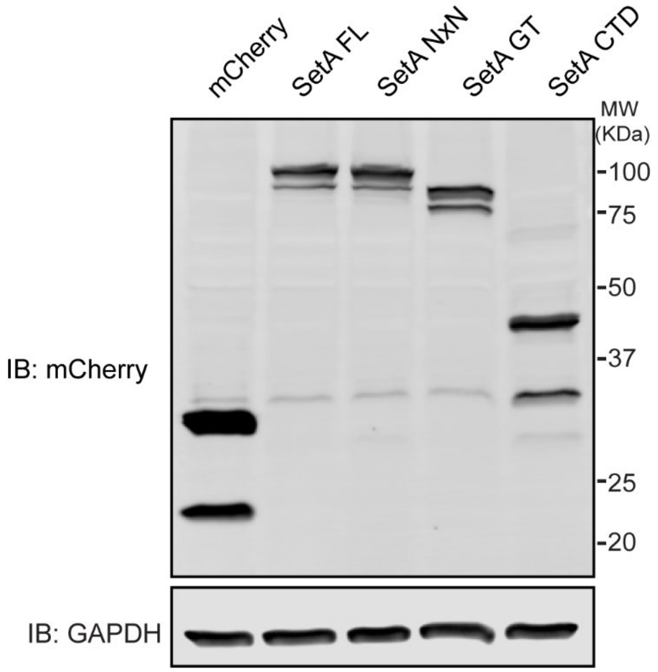

Figure S2, related to Figure 2. Western Blot of mCherry-tagged SetA constructs. HEK293T cells were transfected with mCherry vector or plasmids expressing mCherry tagged SetA constructs. Cell lysates were analyzed by anti-mCherry Western Blot. GAPDH was blotted as a loading control.

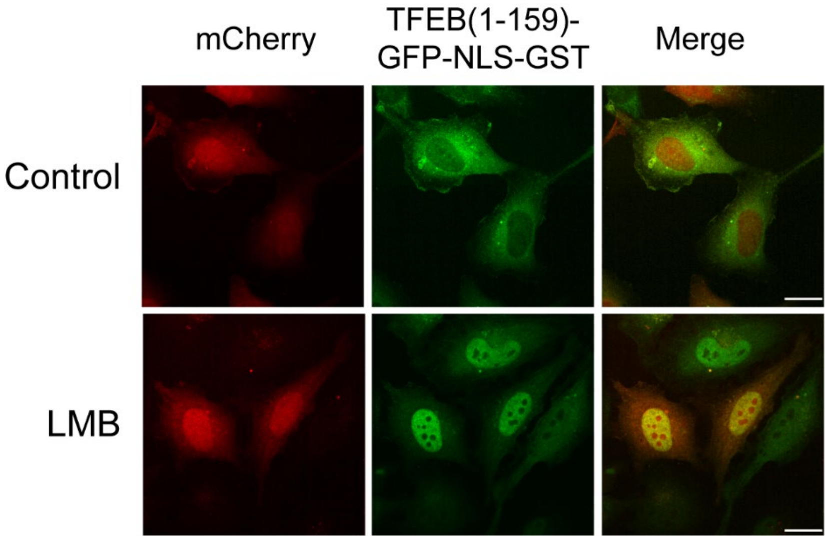

Figure S3, related to Figure 4. Leptomycin B induces nuclear retention of the TFEB-NES-GFP-NLS-cargo reporter. Representative images of a stable TFEB-NES-GFP-NLS-cargo HeLa cell line expressing transfected mCherry vector, untreated (top panel) or treated for 1 hour with 10 nM LMB (bottom panel). A robust nuclear enrichment of the TFEB NES reporter is observed upon treatment with LMB, validating the feasibility of this TFEB-NES-GFP-NLS-cargo reporter expressing cell line for studying TFEB nuclear export. Scale bar represents 20 μm.

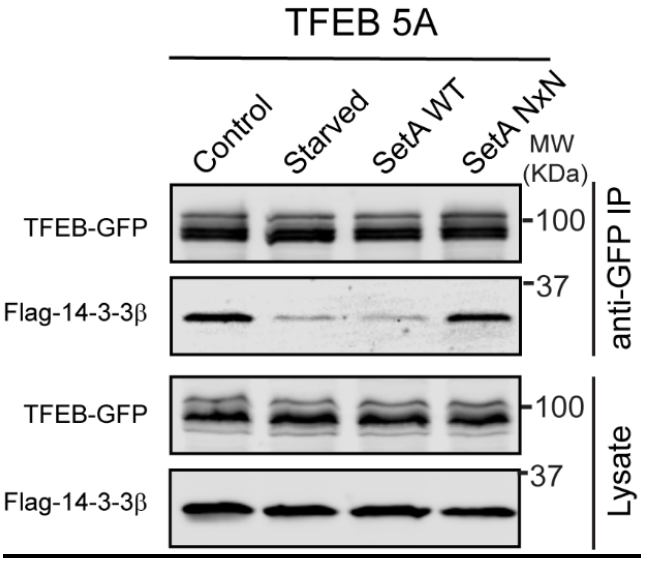

Figure S4, related to Figure 5. TFEB mutant harboring alanine substitutions at all five potential glucosylation sites near S211 remains sensitive to SetA. Western blot analysis of anti-GFP co-IPs from HEK293T cells transfected with plasmids expressing the indicated mCherry-SetA construct, Flag-14-3-3β, and TFEB-GFP with alanine substitutions at S195, S196, T201, S203, and T208 (TFEB 5A). The co-immunoprecipitated Flag-14-3-3β was blotted using an anti-Flag antibody.

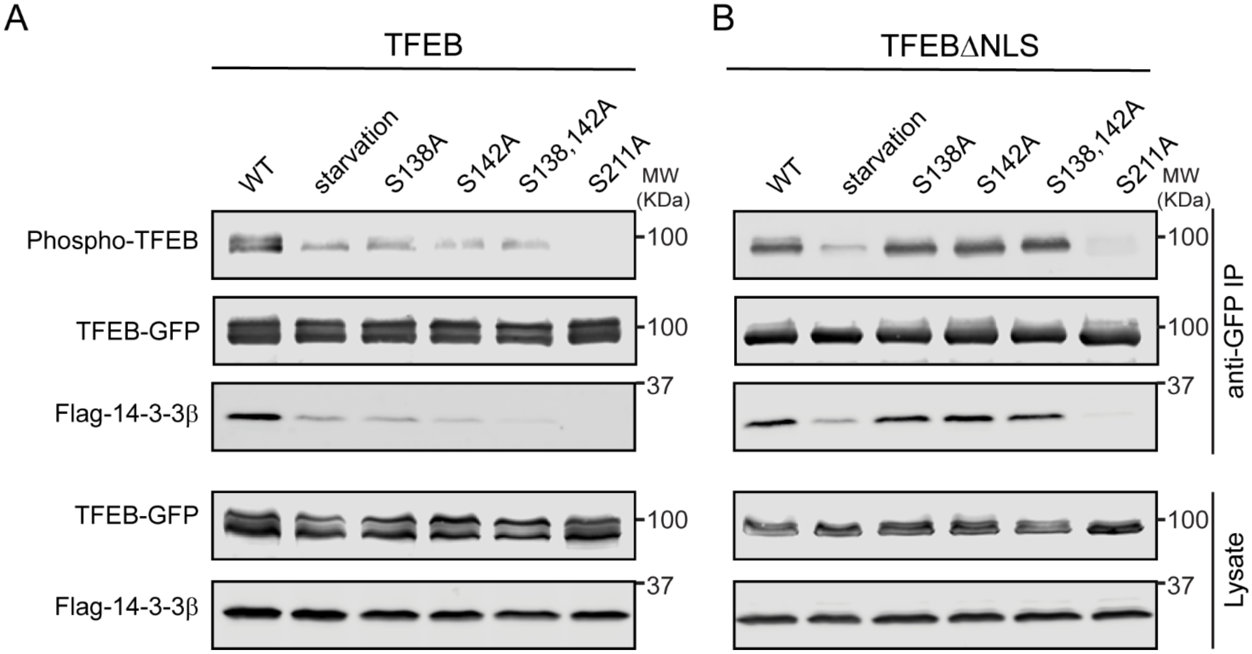

Figure S5, related to Figure 5. Deletion of the NLS restores the binding of 14-3-3 with TFEB mutants carrying mutations at key phosphorylation sites. (A) Western blot analysis of anti-GFP co-IPs from HEK293T cells transfected with plasmids expressing flag-14-3-3β and indicated TFEB-GFP mutants. Immunoprecipitated Flag-14-3-3β was blotted and detected using an anti-Flag antibody and phosphorylation status of TFEB S211 was blotted and evaluated using a phospho-14-3-3 motif antibody. TFEB mutants defective in phosphorylation near the nuclear export signal (S138 and S142) have reduced binding to 14-3-3 and decreased phosphorylation at S211 (phospho-TFEB) (B) Similar Western blot analysis of anti-GFP co-IPs from HEK293T cells transfected with plasmids expressing flag-14-3-3β and TFEBΔNLS-GFP mutants carrying the same alanine substitution on TFEB phosphorylation sites as in (A). Deletion of the nuclear localization signal (NLS) restores phosphorylation at S211 and binding to 14-3-3 even in the presence of S138 and S142 mutations. As a control, alanine substitution at S211 completely disrupts binding to 14-3-3 for both TFEB and TFEBΔNLS in both (A) and (B).

Primer	Sequence (5′ → 3′)
TFEB_deltaNLS_F	caatcacaacttaattgaagcggcagcagcgttcaacatcaatgaccgc
TFEB_deltaNLS_R	gcggtcattgatgttgaacgctgctgccgcttcaattaagttgtgattg
TFEB_wt_S195_196A_F	gcagccacctgaatgtgtacgccgccgacccccaggtcacagcc
TFEB_wt_S195_196A_R	ggctgtgacctgggggtcggcggcgtacacattcaggtggctgc
TFEB_T201A_F:	gcgacccccaggtcgccgcctccctggtggg
TFEB_T201A_R:	cccaccagggaggcggcgacctgggggtcgc
TFEB_S203A_F:	cccaggtcacagccgccctggtgggcgtc
TFEB_S203A_R:	gacgcccaccagggcggctgtgacctggg
TFEB_T208A_F:	ccctggtgggcgtcgccagcagctcctgc
TFEB_T208A_R:	gcaggagctgctggcgacgcccaccaggg
TFEB_T201A_S203A_F	gcagcgacccccaggtcgcagccgccctggtgggcgtcgcc
TFEB_T201A_S203A_R	ggcgacgcccaccagggcggctgcgacctgggggtcgctgc
TFEB_S195_196A_F	gcagccacctgaatgtgtacgccgccgacccccaggtcgcagccg
TFEB_S195_196A_R	cggctgcgacctgggggtcggcggcgtacacattcaggtggctgc

Supplemental Table S1, related to Figure 5. List of mutagenesis primers for TFEB mutant constructs.

TRANSPARENT METHODS

EXPERIMENTAL MODEL AND SUBJECT DETAILS

Cell Culture

HeLa and HEK293T cells were cultured in Dulbecco's Modified Eagle's Medium (DMEM) containing 4.5 g/L glucose, L-glutamine supplemented with 10% fetal bovine serum and 1% penicillin-streptomycin (Invitrogen). HeLa cells stably expressing TFEB-GFP were maintained in 0.5 mg/mL of G418.

Stable Cell Line Generation

HeLa cells stably expressing TFEB (1-159)-GFP-NES-GST reporter constructs were generated using the PiggyBac expression system. HeLa cells were grown to high density (~80-90%) in 6-well plates, and transiently transfected with TFEB NES reporter and the PiggyBac transposase plasmids using Lipofectamine 2000 and Opti-MEM (Invitrogen). Cells were treated with 3 μg/mL of puromycin in D10 media 48 hours post-transfection and maintained for 1 week in the antibiotic for stable cell line selection. Stable cell lines were maintained in 2 μg/mL puromycin thereafter.

METHOD DETAILS

Cloning and Site-Directed Mutagenesis

The SetA (lpg1978) gene was cloned from purified genomic DNA isolated from the *L. pneumophila* Philadelphia 1 strain. All SetA constructs were cloned into a pmCherry-C1 vector using the cut sites BglII/SalI (vector) and BamHI/XhoI (insert). All TFEB mutants used in this study were generated from a pEGFP-N1-TFEB plasmid purchased from Addgene (plasmid # 38119) using site-directed mutagenesis protocols adapted from the Qiagen QuikChange II Site-Directed Mutagenesis Kit All mutagenesis primers used to create the TFEB mutant constructs are listed in Supplemental Table S1.

Legionella Effector cDNA Library

The mCherry-tagged *Legionella* effector library was generated using Gateway cloning techniques. *Legionella* effector genes were PCR amplified from purified genomic DNA isolated from the *L. pneumophilia* Philadelphia 1 strain and ligated into a pDONR221 vector using BP Clonase, to generate the pDONR221 Gateway library. cDNA was subsequently transferred into a mCherry-C1 Gateway destination vector (pDestmCherry-C1) using LR Clonase. *Legionella* cDNA inserts in the mCherry library were verified by PCR.

Legionella Effector Library Screen

For imaging experiments, cells were passaged at ~50% density into 96-well plates (Grenier Bio-One) 24 hours prior to transfection. Plasmids from the mCherry-tagged *Legionella* effector library were transfected at a total amount of 25-50 ng using PEI (1:5 m/v) for 12-16 hours. Cells were fixed in-well using 4% paraformaldehyde then stored in PBS. Fixed cells were imaged using a Zeiss Axio Observer epifluorescence microscope with a 20× long distance 0.4NA objective lens.

Imaging by Confocal Microscopy

Cells were fixed in 4% paraformaldehyde in PBS solution for 20 minutes at room temperature then washed three times in PBS. Fixed coverslips were mounted onto glass slides using Fluoromount-G mounting solution. Fixed cells were imaged using a spinning disk confocal microscope (Intelligent Imaging 108 Innovations, Denver, CO) equipped with a spinning disk confocal unit (Yokogawa CSU-X1), an inverted 109 microscope (Leica DMI6000B), a fiber-optic laser light source, a 40× or 63× 1.47NA objective lens, 110 and a Hamamatsu ORCA Flash 4.0 v2+ sCMOS camera. Images were acquired and processed using the Slidebook (version 6) software.

Co-Immunoprecipitation and Western Blot of TFEB and 14-3-3

TFEB-GFP constructs were transiently co-expressed with Flag-HA-14-3-3β (Addgene plasmid #8999) and pmCherry-SetA or pmCherry-SetA mutant constructs in HEK293T cells. Cells were washed and treated with HBSS or D10 media for14-16 hours post-transfection. After treatment, cells were washed two times with cold PBS and resuspended in 500 μL of IP lysis buffer (1% triton-X, 0.1% deoxycholate in 50 mM tris, 150 mM NaCl, pH 8 containing phosphatase inhibitors and protease inhibitor cocktail (Roche)). Cells were briefly sonicated at 10% amplitude for 5 seconds (pulse) and centrifuged at 15000 rpm for 15 minutes at 4°C to the remove the insoluble fraction. GFP-nanobody conjugated resin was added to the collected supernatant and incubated for 4 hours on a nutating mixer at 4°C to bind TFEB-GFP. Resin containing bound proteins were washed with 1 mL of IP wash buffer (1% triton-X in 50 mM tris, 150 mM NaCl pH 8) for a total of 4 washes. Proteins were eluted from the resin by boiling at 95°C for 4 minutes in 45 μL of SDS sample loading buffer containing 2% BME. Immunoblotting of TFEB-GFP was

performed using a homemade rabbit anti-GFP antibody at a dilution of 1:5000, a gift from Anthony Bretscher (Cornell University). Flag-HA-14-3-3β was probed using mouse anti-Flag M2 clone antibody (Sigma; cat. no. F1804) at a dilution of 1:5000. Probed proteins were detected using donkey anti-rabbit IgG antibody, DyLight 800 (Invitrogen; cat. no. SA5-10044) and donkey anti-mouse IgG antibody, Alexa Fluor 680 (Invitrogen; cat. no. A10038) 2° antibodies. Membranes were scanned using a LI-COR Odyssey CLx Imager. Western Blot images were processed and analyzed using ImageStudio Lite software (version 5.2).

Western Blot Analysis of Phospho-S6K

For phospho-p70 S6 Kinase immunoblotting experiments, mCherry-SetA constructs were transfected into HeLa cells stably expressing TFEB-GFP. Cells were passaged into a 24-well plate at 20% initial density in D10 media. Cells were subsequently transfected 24-hours later with 0.3 µg of plasmid and 1:5 (m/v) ratio of Lipofectamine 2000 in Opti-MEM for a total volume of 50 µL. Media was changed 4 hours post-transfection to remove excess Lipofectamine 2000. Transfection efficiency was assessed 15 hours post-transfection, ranging between 80-90%. Cells were treated with HBSS or D10 media 1 hour prior to sample collection. Cells were washed two times with 500 µL of cold PBS on ice and resuspended in 70 µL of 2X SDS loading buffer containing 10% beta mercaptoethanol (BME). Samples were briefly sonicated at 10% amplitude for 5 sec (pulse) to lyse cells. SDS cell lysate samples (10 µL) were separated on a 12% SDS-PAGE gel. Samples were transferred onto a 0.45 µm pore PVDF membrane for 1 hr at constant 300 milliamps. Membranes were blocked in 5% milk in PBS for 1 hr at room temperature, then washed three times with 0.1% tween in TBS (5-minute washes). Membranes were incubated in 1° antibodies diluted in 0.1% tween in TBS overnight at 4°C. Membranes were washed for 5 minutes

in 0.1% tween in TBS for a total of 3 washes and incubated in 2° antibodies diluted in 0.1% tween in TBS for 2 hours at room temperature. Membranes were washed for 5 minutes in 0.1% tween in TBS for a total of 3 washes and scanned using a LI-COR Odyssey CLx Imager. Western Blot images were processed and analyzed using ImageStudio Lite software (version 5.2).

Total S6K was probed using a rabbit α P70 S6K was purchased from ProteinTech (cat. no. 14485-1-AP) at a dilution of 1:1000. Phospho-S6K was probed using rabbit mAb anti-phospho-p70 S6 Kinase (Thr389) (108D2) antibody purchased from Cell Signaling at a dilution of 1:1000. Total GAPDH levels were probed using a mouse anti-GAPDH from ProteinTech (cat. no. 60004) at a dilution of 1:5000. Donkey anti-rabbit IgG antibody, DyLight 800 (Invitrogen; cat. no. SA5-10044) and donkey anti-mouse IgG antibody, Alexa Fluor 680 (Invitrogen; cat. no. A10038) 2° antibodies were used for Western Blot imaging.

SILAC Labeling and Sample Preparation

HEK293T cells were grown in complete media containing normal lysine and arginine ("light") or [13C6,15N2] lysine and [13C6,15N4] arginine ("heavy", Sigma) for a minimum of 5 passages prior to transfection to ensure complete labeling. The labeled cells were passaged at 20% density into 10 cm dishes. TFEB-GFP and Flag-HA-14-3-3β were co-expressed with wild-type SetA (mCherry-SetA 1-506; C13 heavy) or the catalytic-dead version of SetA (mCherry-SetA NxN 1-506; C12 light) in HEK293T cells for 16 hours. Cells were then washed two times with cold PBS and collected with a lysis buffer (1% triton-X 100 in 50 mM tris, 150 mM NaCl pH 8, 0.1% deoxycholate, phosphatase inhibitor cocktail, and protease inhibitor (Roche)). Cells were briefly sonicated (10% amplitude, 5 sec duration, pulse) and centrifuged at 14000 rpm for 15 minutes at 4°C to remove the insoluble fraction. Supernatant was collected and mixed with 30 μL

slurry of homemade GFP-nanobead conjugated resin (TFEB-GFP) or EZ-view Red anti-Flag M2 resin (Flag-HA-14-3-3β). The mixture was incubated on a nutating mixer at 4°C for 4 hrs. Resin containing bound proteins were spun down and subsequently washed with washing buffer (1% triton-X in 50 mM tris, 150 mM NaCl pH 8.0) for a total of 5 washes. Bound proteins were eluted by incubating of the resin with 45 μL of elution buffer containing 1% SDS in 100 mM tris pH 8.0 at 65°C for 15 minutes. Eluted proteins from light or heavy media grown cells were mixed together, reduced, alkylated with iodoacetamide and then precipitated with three volumes of a solution containing 50% acetone and 50% ethanol. Precipitated proteins were solubilized in a solution of 2 M urea, 50 mM tris-HCl, pH 8.0, and 150 mM NaCl, and then digested with Pierce trypsin protease MS-grade (Thermo Scientific) overnight at 37°C. Digested peptides were acidified with 0.2% Trifluoroacetic acid and formic acid and then desalted with Sep-Pak C18 column (Waters) for mass spectrometry analysis.

Mass Spectrometry Data Acquisition and Analysis

Desalted peptides were dried, resuspended in 0.1% trifluoroacetic acid and injected into a QExactive Orbitrap mass spectrometer (Thermo Fisher Scientific) using 20-cm long in-house packed column. Raw MS/MS spectra were searched using the SORCERER (Sage N Research, Inc.) system running the SEQUEST software over the human Uniprot proteome database. Searching parameters included a semi-tryptic requirement, a mass accuracy of 15 ppm for the precursor ions, differential modification of 8.0142 daltons for lysine, 10.00827 daltons for arginine, 162.05279 daltons for glucosylation of serine and threonine, and a static mass modification of 57.021465 daltons for alkylated cysteine residues. XPRESS software, part of the Trans-Proteomic Pipeline (Seattle Proteome Center), was used to quantify all the identified

peptides. MS/MS spectra for TFEB peptides were manually inspected and interpreted for defining proper identification and glucosylation site position.

QUANTIFICATION AND STATISTICAL ANALYSIS

Quantification and statistical analysis for TFEB-GFP nuclear translocation experiments represents three independent replicates ($n = 3$) with a minimum of 100 cells counted per construct in each experiment. Quantification and statistical analysis for the TFEB (1-159)-GFP-NLS-GST reporter experiments represents four independent replicates ($n = 4$) with a minimum of 50 cells counted per construct in each experiment. Quantification of relative 14-3-3 pulldown levels represents three independent replicate experiments ($n = 3$). Error bars represent standard error of the mean (SEM) values. Significance was calculated using an unpaired two-tailed t-test with unequal variance. Statistical significance of $p < 0.05$ or lower is reported on analyzed data sets. All statistical analyses were performed using GraphPad Prism (version 6).
